# Randomized, Double-Blind Clinical Trial to Assess the Acute Diuretic Effect of *Equisetum arvense* (Field Horsetail) in Healthy Volunteers

**DOI:** 10.1155/2014/760683

**Published:** 2014-03-04

**Authors:** Danilo Maciel Carneiro, Ramias Calixto Freire, Tereza Cristina de Deus Honório, Iury Zoghaib, Fabiana Fernandes de S. e Silva Cardoso, Leonice Manrique F. Tresvenzol, José Realino de Paula, Ana Luiza Lima Sousa, Paulo César Brandão Veiga Jardim, Luiz Carlos da Cunha

**Affiliations:** ^1^Group of Toxicopharmacological Studies and Research, School of Pharmacy, Federal University of Goias, Avenida Universitária, Q. 62, Setor Universitário, 74605-220 Goiânia, GO, Brazil; ^2^Hospital of Alternative Medicine, State Health Secretary of Goiás, Unified Health System, Rodovia BR-153, Km 8, Bairro Santo Antônio, 74853-040 Goiânia, GO, Brazil; ^3^Romulo Rocha Center of Clinical Analysis, School of Pharmacy, UFG, Avenida Universitária c/1a Avenida, Q. 62, Setor Universitário,74605-220 Goiânia, GO, Brazil; ^4^Institute of Pharmaceutical Sciences, Rua João de Abreu Esquina c/Rua 09 No. 192 Qd. F-8, Lt. 24E, Sala A-141 a A-147, Setor Oeste, 74175-100 Goiânia, GO, Brazil; ^5^Arterial Hypertension League of Federal University of Goias, School of Medicine of the Federal University of Goias (UFG), Rua 235 c/1a Avenida s/n, Setor Universitário, 74605-220 Goiânia, GO, Brazil; ^6^School of Nursing of the Federal University of Goias (UFG), Rua 235 c/1a Avenida, s/n, Setor Universitário, 74605-220 Goiânia, GO, Brazil; ^7^School of Medicine of the Federal University of Goias (UFG), Rua 235 c/1a Avenida, s/n, Setor Universitário, 74605-220 Goiânia, GO, Brazil

## Abstract

In this double-blind, randomized clinical trial, 36 healthy male volunteers were randomly distributed into three groups (*n* = 12) that underwent a three-step treatment. For four consecutive days, we alternately administered a standardized dried extract of *Equisetum arvense* (EADE, 900 mg/day), placebo (corn starch, 900 mg/day), or hydrochlorothiazide (25 mg/day), separated by a 10-day washout period. Each volunteer served as his own control, and the groups' results were compared. We repeated the same evaluation after each stage of treatment to evaluate the safety of the drug. The diuretic effect of EADE was assessed by monitoring the volunteers' water balance over a 24 h period. The *E. arvense* extract produced a diuretic effect that was stronger than that of the negative control and was equivalent to that of hydrochlorothiazide without causing significant changes in the elimination of electrolytes. There was no significant increase in the urinary elimination of catabolites. Rare minor adverse events were reported. The clinical examinations and laboratory tests showed no changes before or after the experiment, suggesting that the drug is safe for acute use. Further research is needed to better clarify the mechanism of diuretic action and the other possible pharmacological actions of this phytomedicine.

## 1. Introduction

Interest in phytotherapeutic agents is growing in North America and Europe [1]. The World Health Organization (WHO) reports that treatments using medicinal plants are highly lucrative internationally, and more than 100 countries have established regulations for phytotherapeutic products. The annual profits of this business reached 5 billion USD in Western Europe between 2003 and 2004, 14 billion USD in China in 2005, and 1.1 billion BRL in Brazil in 2011 [2, 3]. *Equisetum arvense *is from the family Equisetaceae, and it is popularly known as “cavalinha” in Brazil and “horsetail” in North America and Europe. *Equisetum arvense *L. is one of the most widely prescribed medicinal plants. This plant is found across the Americas, Europe, Northern Africa, and Asia [4–7], and it is traditionally used as a diuretic and a remineralization, antiedematous, and anti-inflammatory agent [8–10].

The aerial parts of *Equisetum arvense *contain flavonoids, saponins, caffeic acid, phenolic compounds, alkaloids, sterols, and minerals (primarily silicon and potassium salts). The presence of high concentrations of flavonoids, phenolic compounds, and mineral salts indicates a mild diuretic action of this herbal medicine. The abundance of silicon salts indicates that *Equisetum arvense *may possess remineralization properties [11, 12].

Preclinical studies have revealed various pharmacological actions of *Equisetum arvense*, including antioxidant [11, 13] and antidiabetic [14, 15] properties, but no acute hepatotoxicity [16].

The health regulatory agencies of the Federal Republic of Germany established the German Commission E in 1974. This commission approved the use of *Equisetum arvense *for the treatment of posttraumatic and static edema and as a diuretic for bacterial and inflammatory diseases of the urinary tract presenting with urinary sediment [8]. Topical use as an adjuvant for the treatment of wounds that exhibit difficult healing has been reported [8, 17, 18]. However, *E. arvense *does not satisfy the requirements as a well-established medicine, despite an ancient tradition of use because clinical studies of its effects on renal function and safety are lacking. The “Assessment of Medicinal Products for Human Use” of the European Medicines Agencies concluded that clinical data on the absorption, distribution, and pharmacokinetics of *Equisetum arvense *are scarce or completely lacking [8, 19].

The present study assessed the diuretic action and the short-term safety of a standardized dry extract of the aerial parts of *Equisetum arvense *in healthy men using a randomized, double-blind clinical study. The effects of the extract on fluid balance (FB) and urinary catabolite excretion, as well as its possible toxic effects on liver, kidney, and heart function, were assessed. The drug effects were compared to those of a placebo (corn starch) and a classic diuretic (hydrochlorothiazide).

## 2. Materials and Methods

### 2.1. Study Materials

Three compounds were prepared: a standardized dry extract of the aerial parts of *E. arvense *containing 0.026% total flavonoids, hydrochlorothiazide, and placebo (starch). These compounds were formulated by the Farmácia Artesanal (Artesanal Pharmacy), a compounding pharmacy that is duly licensed and certified by the official regulatory agencies of the city of Goiânia (Goiás, Brazil) and that possesses the appropriate analytical and quality assurance certificates. The authenticity of the *E. arvense *sample used in the present study was assessed by the Laboratory of Natural Product Research (Laboratório de Pesquisa de Produtos Naturais (LPPN)) of the School of Pharmacy of the Federal University of Goiás (Universidade Federal de Goiás (UFG)) using thin-layer chromatography following the method described by Wagner and Bladt [20].

The tested substances were placed in capsules, which were placed in vials and labeled with the letters A, B, and C. One of the investigators established this code, which remained secret until the statistical analyses were complete.

### 2.2. Selection of Volunteers

The clinical trial complied with the guidelines of Resolution 196/96 of the National Health Council and the Declaration of Helsinki for research on human beings. The research ethics committee of UFG, which is accredited by the National Commission of Research Ethics of the National Health Council/Health Ministry, approved the protocol (no. 312/10).

The volunteers completed an informed consent form that granted the researchers the right to use the collected materials to perform the required tests to establish the correct medication use, as described in the experimental protocol.

The Recruitment Department of the Institute of Pharmaceutical Science (Instituto de Ciências Farmacêuticas—ICF) recruited and selected all of the volunteers using an institutional database of volunteers who were duly oriented to the importance of appropriate and reliable data collection. Furthermore, the records of these volunteers indicated that the results of biochemical screening tests three months before the onset of the study had been normal.

A total of 36 healthy male volunteers who were 20 to 55 years of age, 1.50 to 1.85 m tall, and 50 to 90 kg in weight were selected on the basis of their medical histories, past medication use, physical examinations, vital signs, and laboratory results.

Smokers and individuals with congestive heart failure, arterial hypertension, or a history of kidney disease, cardiovascular diseases, endocrine disorders, or other underlying diseases were excluded.

The other exclusion criteria included a lack of proper compliance with the protocol at any stage of the study and the appearance of unexpected or exacerbated reactions.

### 2.3. Experimental Protocol

The 36 volunteers (numbered 1 to 36) were randomly allocated to six different drug administration sequences (*n* = 6), in accordance with the design for practical use suggested by Williams (1949), which allows the detection of possible differences between drug treatments [21, 22]. The volunteers were subjected to a three-step treatment protocol in which the order of the three treatments (i.e., *Equisetum arvense, *hydrochlorothiazide, and placebo) was alternated. Each treatment was administered for four consecutive days at each stage, and there was a 10-day washout interval between treatment stages. All of the volunteers consumed one capsule three times per day during each stage of the treatment, followed by the washout period, during which the volunteers received no treatments but continued to follow the diet and lifestyle recommendations (see below). All of the volunteers underwent all three treatments and served as their own controls. The treatment regimen was repeated under the same methodological conditions across the three stages of the study (Table 1).

#### 2.3.1. Definition of the Study Time Points

Day 0 corresponded to the day before treatment onset. Days 1, 2, 3, and 4 were the treatment days, which were followed by the 10-day washout period. Therefore, day 10 of any washout period was also day 0 of the following stage.

The volunteers recorded their fluid input and urinary output over 24 h (24 h fluid balance) on day 0 and during the four days of the treatment to assess the effects of the treatments. On day 0 and the day immediately following the end of each treatment stage, the volunteers underwent the medical assessments and laboratory tests that were defined in the protocol.

### 2.4. Drug Treatment

The capsules used in all the treatments exhibited an identical appearance (size 0 gel capsules). The volunteers received two vials, labeled Vial 1 and Vial 2, at each treatment stage. Vial 1 contained four blue capsules to be taken once per day in the morning for four days, and Vial 2 contained eight capsules to be taken twice per day for four days. The capsules were delivered randomly to the volunteers at the Clinical Research Unit of ICF.

*(i) Treatment A (Equisetum arvense). *Vial 1 contained four blue capsules, and Vial 2 contained eight green capsules. All of the capsules contained 300 mg *E. arvense *(a dry extract with 0.026% total flavonoids). The daily dose of 900 mg was divided into three daily doses of one capsule each. 

*(ii) Treatment B (Hydrochlorothiazide).* Vial 1 contained four blue capsules of 25 mg hydrochlorothiazide, and Vial 2 contained eight green capsules of 300 mg starch (placebo). The total daily dose of hydrochlorothiazide was a single, 25 mg dose taken in the morning, and two doses of placebo were taken daily.

*(iii) Treatment C (Starch).* Vial 1 contained four blue capsules, and Vial 2 contained eight green capsules. All of the capsules contained 300 mg starch. The volunteers took one capsule three times per day.

### 2.5. Laboratory Examinations

All the volunteers underwent blood and urine tests. The blood tests included a complete blood count and assessments of the concentrations of fasting blood glucose, albumin, aspartate transaminase (AST), alanine transaminase (ALT), gamma-glutamyl transpeptidase (GGT), total bilirubin, direct and indirect bilirubins, urea, creatinine, sodium, potassium, chlorine, phosphorus, and magnesium. The urine tests included a urinalysis and analyses of the sodium and potassium levels.

The blood and urine samples were collected at the Clinical Research Unit of ICF by pharmacists who were affiliated with the Group of Toxicopharmacological Studies and Research of UFG (NEPET-UFG). All the samples were appropriately packaged and were transported to the Rômulo Rocha Laboratory of the School of Pharmacy of UFG for analysis. The samples were collected exclusively on the day before treatment onset and the day after the end of treatment for each stage of the study.

All the volunteers underwent two ECG tests: the first test before the onset of the study and the second immediately following the end of the study.

### 2.6. Medical Assessments and Treatment Prescriptions

The medical assessments were performed and the treatments were prescribed in the Clinical Research Unit of ICF. The complete clinical history of each volunteer was obtained at the beginning of the study, and a clinical assessment was conducted at each stage of the study prior to and after the treatments to record clinical manifestations, possible adverse reactions, and physical examination results.

### 2.7. Activities during the Experimental Phase

The volunteers were provided with guidelines for their lifestyle, diet, and physical activity before the onset of the experimental phase. The volunteers were requested not to change their diet during the study and to avoid drug use, alcoholic drinks, and beverages with a diuretic effect (e.g., coffee and tea). The volunteers were also asked to perform their usual physical and daily routine activities and avoid more intense or long-lasting exercises than usual.

### 2.8. Recording of Fluid Input and Output and Fluid Balance

The volunteers were provided with backpacks to carry continually throughout the study. Each backpack included two separate compartments to store (in a hygienic manner) a graduated measuring cup to measure fluid intake, a vessel with a cap to measure urine, a notebook, and a pen to record the volumes of ingested fluids and urination. All of the volunteers signed a form indicating the receipt of the materials and their commitment to comply with the provided instructions. From day 0 to day 4, the volunteers recorded the volume of urine after each urination and, immediately after any intake of fluid, the volume of fluid ingested. These data served as the basis for the fluid balance (FB) calculations.

Therefore, the FB represented the difference between the volumes of fluid intake and excreted urine over 24 h, from the first urination in the morning after awakening to the final urination before arising from bed the following morning [23].

### 2.9. Assessment of Diuretic Effects

The diuretic effects were assessed using the following criteria.

- Assessment of the intragroup (A, B, and C) final FB: the final FB corresponded to the difference between the posttreatment FB and the initial FB (without treatment; FB0). Therefore, final FB = posttreatment FB − FB0.
- Assessment of the intergroup (A, B, and C) final FB: the final FBs were compared between the groups.
- Assessment of the effects of the treatments on urine electrolyte concentrations: the results of the measurements on day 4 were compared to the results on day 0 at each stage of the experiment.

### 2.10. Assessment of Safety

#### 2.10.1. Reporting of Adverse Events

The volunteers were asked to report any symptoms and the use of any emergency medications. A researcher recorded all the reported adverse events during the clinical trial on case report forms (CRFs) for clinical followup. The reported symptoms were classified on the basis of their intensity: mild, moderate, or severe [24].

#### 2.10.2. Additional Tests

Possible acute toxic effects related to liver, kidney, and heart functions were investigated during the clinical and laboratory examinations that were performed before and after the treatments at each stage of the study. ECGs were performed at the onset and conclusion of the study.

### 2.11. Methodological Control Measures

The following resources were used to reduce and monitor noncontrolled parameters, such as fluid and sodium intake, physical activity, and the effects of other drugs.

1. The study included volunteers who were aware of the importance of clinical research and who were recruited, selected, and oriented by an independent institute of clinical research with experience in clinical trials (ICF, Goiânia).
2. The volunteers were asked to maintain their usual diet and exercise routines and to avoid the use of products and physical activities that might interfere with the study results.
3. The volunteers were contacted daily by telephone during the three stages of medication use, and they underwent weekly clinical assessments throughout the 42-day study period.
4. The volunteers received orientation at recruitment meetings and in a written format.
5. The calculation of the actual fluid balance, instead of the controlled volume of fluid intake minus the urine volume, represents an organism's global response to real-life factors that controlled studies do not consider. However, commercially available drugs do consider this global response.

### 2.12. Measures to Ensure the Relevance and Reliability of the Collected Data

The following measures were applied to determine the variability of the noncontrolled real-life variables between the individuals and groups, which might have interfered with the study results.

- Statistical tests were used to assess the groups' homogeneity relative to the FB0 at each stage and to assess the reliability of this parameter for the assessment of diuretic activity.
- A crossover design and the Williams design for randomization were used to reduce the impact of the noncontrolled variables and to increase the data reliability.

### 2.13. Data Analysis

The data were stored in Microsoft Excel for Windows for later analysis with the *Statistical Package for the Social Sciences *(SPSS), version 17.0.

The Kolmogorov-Smirnov test was used to establish the normality of the data distribution.

A paired Student's *t*-test was used to investigate the significance of differences in the normally distributed data prior to and after treatment. Student's *t*-test for independent samples was used to investigate the presence of significant differences between treatments A, B, and C.

An analysis of variance was used to evaluate the significance of differences between the variables for treatments A, B, and C.

Wilcoxon's test and Pearson's correlation test were used to analyze the volunteers' urinary sodium and potassium concentrations prior to and after the treatments.

The confidence level was established as 95% (i.e., *P* < 0.05).

## 3. Results and Discussion

### 3.1. Analysis of the Dry Extract

Laboratory tests confirmed that the dry extract used in the study corresponded to *E. arvense*. This analysis was performed using thin-layer chromatography (TLC) according to Wagner and Bladt [20].

### 3.2. Investigated Sample

The individuals in the various treatment groups did not exhibit significant differences in age (*P* = 0.872), weight (*P* = 0.768), height (*P* = 0.489), or body mass index (BMI) (*P* = 0.980), which confirms the homogeneity of the groups at the beginning of the study.

### 3.3. Fluid Balance (FB)

The FB values of all the volunteers on day 0 (FB0) were compared at each stage of the study. The FB0s were not different between the individual volunteers or between the groups that were established using the Williams design. A comparison of the FB0s of groups A, B, and C revealed no significant intergroup differences (*P* > 0.05). Similarly, the FB0 values of all the studied groups exhibited similar behaviors at the three stages of data collection.

The fluid intake volumes of the volunteers were similar in all three stages of the experiment. The volume of urine, as a whole, also did not differ between the three data collection stages (*P* > 0.05).

These data support the homogeneity of the volunteers and confirm the reliability of the FB0 value as a reference parameter.

The FB0 values exhibited a normal distribution according to the Kolmogorov-Smirnov test, despite demonstrating a wide variation. This result indicated that the FB would be highly variable even among individuals of homogeneous age, weight, height, and BMI.

### 3.4. Diuretic Effect

The diuretic effects of the treatments were assessed using the following criteria, according to the parameters in Section 2.9.

*(a) Assessment of the Intragroup Final FB.* Group A (*E. arvense*) exhibited a negative final FB of −321.81 ± 481.02 mL (*P* < 0.001). Group B (hydrochlorothiazide) exhibited a final FB of −231.84 ± 726.60 mL, which was not significantly different from those of the other groups (*P* = 0.067). Group C (placebo) exhibited a positive final FB of 130.27 ± 534.30 mL, which was not significantly from those of the other groups (*P* = 0.164). A negative final FB is an important criterion for establishing the presence of diuresis. Therefore, a negative final FB indicated the induction of a diuretic effect by *E. arvense *and hydrochlorothiazide and the absence of this effect with placebo [25] (Table 2). 

**Figure 1:**
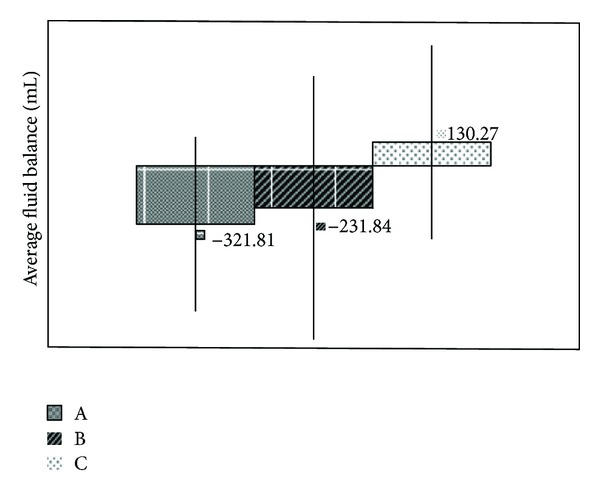
Comparisons of the fluid balance between groups (*n* = 36/group). Groups: A = *Equisetum arvense*; B = hydrochlorothiazide; C = placebo. A versus C (*P* < 0.001). B versus C (*P* = 0.026). A versus B (*P* = 0.056).

*(b) Assessment of the Intergroup Final FB.* Intergroup comparisons of the final FBs revealed significant differences between Groups A (*E. arvense*) and C (placebo) (*P* < 0.001) and between Groups B (hydrochlorothiazide) and C (placebo) (*P* = 0.026). However, no difference between Groups A and B was observed (*P* = 0.566). These results demonstrate that *E. arvense *and hydrochlorothiazide were associated with similar, negative final FB values, which suggests that these agents produced a similar diuretic effect that was superior to that of the placebo (Figure 1). 

*(c) Assessment of the Effects of the Treatments on Urinary Electrolyte Concentrations*. The rates of urinary sodium and potassium excretion were not significantly altered by the treatments at any of the three stages of the study. Pearson's correlation revealed no correlations between FB and sodium excretion during the first (*P* = 0.513), second (*P* = 0.824), or third (*P* = 0.513) stages of the study. Similarly, no correlations between FB and potassium excretion were observed during the first (*P* = 0.388), second (*P* = 0.425), or third (*P* = 0.931) stage.

The negative final FB of hydrochlorothiazide was similar to that of *E. arvense*, which supports the existence of a diuretic effect of *E. arvense *[25]. However, the large standard deviations of the final FB values may have negatively affected the significance of the diuretic effect in the *E. arvense *group. This statistical result was caused by the wide heterogeneity of the volunteers' responses following hydrochlorothiazide administration. This result is not surprising; previous studies have reported variability in the response to this drug. Earley and Orloff [26] and Kim et al. [27] discussed the antidiuretic effects of hydrochlorothiazide in patients with diabetes insipidus. One clinical trial suggested that the observed variations in the pharmacokinetics of hydrochlorothiazide were functions of individual sensitivities to this drug and the presence of digestive disorders that may have impaired drug absorption [28]. Another study assessed the control of arterial pressure using hydrochlorothiazide. These authors reported such wide variations in the magnitude and direction of the response between the volunteers that they considered the measurement of blood pressure at medical offices to be inadequate for predicting arterial pressure control in an outpatient setting. However, these authors attributed the observed variability to discrepancies between the measuring techniques [29].

The dose of *E. arvense *administered in the present work was the maximum recommended dose for dry extracts (900 mg) [25]. The use of this dosage might explain the comparable diuretic effects of *E. arvense *and hydrochlorothiazide at a daily dose of 25 mg. The results concerning the diuretic effect of *E. arvense *corroborate some previous preclinical data. The European Medicines Agency has demonstrated a diuretic effect of *E. arvense *in dogs, and common horsetail is an effective and fast-acting diuretic agent in rats that does not induce a negative FB. Kreitmair (1936 and 1953) and Herre (1937) demonstrated results similar to ours. Volmer (1937, 1939, 1940, and 1941) used an *E. arvense *tea to demonstrate diuretic effects and negative FBs in rats and rabbits (as cited in [25]). Rebuelta and San Roman [30] observed a mild diuretic effect of *E. arvense *in a preclinical study.

Our experimental data confirm the ethnopharmacology and traditional use of *E. arvense *as a diuretic, which occurs worldwide, including in Brazil and other South American countries [5, 10, 16–18, 25, 30] and in several European [4, 6–9, 11, 25] and Asian countries [4, 13–15, 24]. These results represent a major stimulus for further clinical studies of the other pharmacological effects of *E. arvense *and other medicinal plants with broad scopes in traditional medicine.

### 3.5. Usage Safety

#### 3.5.1. Additional Tests

The results of the additional tests (Section 2.10.2) revealed that the values of all of the investigated variables remained within the normal ranges, which suggests that no acute manifestations of kidney, liver, hematological, or electrolyte toxicity occurred. The results of Student's *t*-test revealed significant variations in some of the assessed values, but none of these results exhibited values outside of the normal range. During stage 1, *E. arvense *induced significant reductions in the creatinine (*P* = 0.003) and uric acid (*P* = 0.010) levels. During stage 2, *E. arvense *induced significant reductions in the ALT (*P* = 0.022), GGT (*P* = 0.007), and phosphorus (*P* < 0.001) levels and significant increases in the urea (*P* = 0.025) and creatinine (*P* = 0.006) levels. During stage 3, *E. arvense *induced significant reductions in the GGT (*P* = 0.006), chloride (*P* = 0.042), magnesium ion (*P* = 0.044), and phosphorus (*P* = 0.032) levels.

The results of the laboratory tests remained within the normal ranges for all three stages of the study prior to and after treatment. No signs of liver, kidney, hematological, or electrolyte toxicity were identified.

The volunteers in Group A exhibited consistent reductions in GGT during the second and third stages (*P* = 0.007 and 0.006, resp.), which is consistent with the use of *E. arvense *in traditional Chinese medicine and its possible liver protective action [31]. Reductions in ALT during the second treatment stage were also observed in Group A.

#### 3.5.2. Adverse Effects

All the treatments were well tolerated overall. The few symptoms that occurred exhibited a homogeneous distribution across all three groups. One volunteer complained of a strong headache during the first stage of *E. arvense *treatment. One volunteer reported a strong, adverse reaction consisting of copious diuresis and polydipsia following a single dose of hydrochlorothiazide during the first stage of the study. Two volunteers reported irregular compliance with the protocol. The latter three volunteers were removed from the study and were immediately replaced with other volunteers from the same ICF volunteer database using the same inclusion and exclusion criteria. The rest of the reported symptoms were classified as mild. Most of the symptoms improved without the use of symptomatic medication, and only two volunteers used analgesics (acetaminophen) during the experiment. Headache was the most frequently reported symptom; it occurred with all of the treatments at most stages of the study.

### 3.6. Arterial Blood Pressure

The systolic pressure was significantly reduced after treatment compared to pretreatment levels only at the first stage in the group that underwent the BCA sequence (*P* = 0.037). No significant differences before and after treatment were observed in the other groups at any of the three stages.

## 4. Conclusions

*E. arvense *produced a diuretic effect as assessed with FB measurements. This effect was comparable to that of hydrochlorothiazide (25 mg) and was superior to that of placebo (starch).

*E. arvense *did not exert significant effects on the urinary excretion of electrolytes and catabolites, and it was deemed safe for oral use. No alterations were observed in the liver, kidney, or hematological function tests, and the reported adverse reactions were mild and infrequent.

Future studies are required to elucidate the mechanism of the diuretic action of *E. arvense *and the other possible pharmacological actions of this phytomedicine.

## Figures and Tables

**Table 1:** Drug administration sequences of study according to the proposed design by Williams.

	Stage				
	First		Second		Third
	Treatment: 4 days	10 days	Treatment: 4 days	10 days	Treatment: 4 days
Seq. 1 ABC	A (*n* = 6)	Washout	B (*n* = 6)	Washout	C (*n* = 6)
Seq. 2 CAB	C (*n* = 6)	Washout	A (*n* = 6)	Washout	B (*n* = 6)
Seq. 3 BCA	B (*n* = 6)	Washout	C (*n* = 6)	Washout	A (*n* = 6)
Seq. 4 CBA	C (*n* = 6)	Washout	B (*n* = 6)	Washout	A (*n* = 6)
Seq. 5 BAC	B (*n* = 6)	Washout	A (*n* = 6)	Washout	C (*n* = 6)
Seq. 6 ACB	A (*n* = 6)	Washout	C (*n* = 6)	Washout	B (*n* = 6)

**Table 2:** Intragroup comparisons of fluid balance (*n* = 36/group). The values represent the means ± SD, and the significance was set at *P* < 0.05.

Group	Fluid Balance			
	FB0 (mL)	FB (mL)	FFB (mL)	*P *
A	844.43 ± 632.72	522.62 ± 463.03	−321.81 ± 481.02	<0.001*
B	773.86 ± 955.37	542.01 ± 935.37	−231.84 ± 726.60	0.067
C	634.69 ± 511.46	763.21 ± 482.45	130.27 ± 534.30	0.164

Groups: A: *Equisetum arvense*; B: hydrochlorothiazide; C: placebo. FB0: initial fluid balance without treatment; FB: FB after treatment; FFB: final fluid balance (FB − FB0).

*P < 0.05.
